# Development of nanoparticle-based optical sensors for pathogenic bacterial detection

**DOI:** 10.1186/s12951-017-0260-y

**Published:** 2017-03-31

**Authors:** Teodora Mocan, Cristian T. Matea, Teodora Pop, Ofelia Mosteanu, Anca Dana Buzoianu, Cosmin Puia, Cornel Iancu, Lucian Mocan

**Affiliations:** 1 Department of Nanomedicine, “Octavian Fodor” Gastroenterology Institute, 19-21 Croitorilor Street, Cluj-Napoca, Romania; 2 Department of Physiology, “Iuliu Hatieganu” University of Medicine and Pharmacy, 3-5 Clinicilor Street, Cluj-Napoca, Romania; 3 3rd Gastroenterology Department, “Iuliu Hatieganu” University of Medicine and Pharmacy, 19-21 Croitorilor Street, Cluj-Napoca, Romania; 4 Department of Clinical Pharmacology, “Iuliu Hatieganu” University of Medicine and Pharmacy, 3-5 Clinicilor Street, Cluj-Napoca, Romania; 5 3rd Surgery Clinic, “Iuliu Hatieganu” University of Medicine and Pharmacy, 19-21 Croitorilor Street, Cluj-Napoca, Romania

**Keywords:** Bacteria, Detection assay, Nanoparticles, Antibiotic resistance

## Abstract

**Background:**

Pathogenic bacteria contribute to various globally important diseases, killing millions of people each year. Various fields of medicine currently benefit from or may potentially benefit from the use of nanotechnology applications, in which there is growing interest. Disease-related biomarkers can be rapidly and directly detected by nanostructures, such as nanowires, nanotubes, nanoparticles, cantilevers, microarrays, and nanoarrays, as part of an accurate process characterized by lower sample consumption and considerably higher sensitivity. There is a need for accurate techniques for pathogenic bacteria identification and detection to allow the prevention and management of pathogenic diseases and to assure food safety.

**Conclusion:**

The focus of this review is on the current nanoparticle-based techniques for pathogenic bacterial identification and detection using these applications.

## Background

Pathogenic bacteria contribute to various globally important diseases, killing millions of people each year [1]. The amount of pathogen required to cause infection in the host does not need to be high to lead to life-threatening conditions such as sepsis, which is a major public health concern [2]. The incidence of sepsis is underestimated, and common risk factors include being of a very young or old age. A drawback in the management of sepsis is the length of time required to develop cultures for diagnosis. A severe blood infection, sepsis requires the identification of the responsible organism (bacterium, fungus, virus, or parasite). When bacteria are present, a more thorough analysis involves extensive cell cultures. Sepsis is only treated after identification of the microorganism and assessment of its antibiotic resistance [3]. Bacterial counts below 10 colony-forming units per milliliter (CFU/mL) of blood are not sufficient for identifying bacteria using non-DNA based techniques; only additional bacterial cultures can provide the amount necessary for accurate diagnosis [4].

Various fields of medicine currently benefit from or may potentially benefit from the use of nanotechnology applications, in which there is growing interest [5–8]. The use of nanotechnology in medicine, especially in drug delivery, may revolutionize this domain and is expected to spread rapidly in coming years [9–16]. Various other applications are based on genetic engineering advances and improved imaging technologies, such as in vivo imaging, in vitro diagnostics (which can detect diseases, conditions, or infections), multiple types of therapies, biomaterials (which interact with biological systems for therapeutic or diagnostic purposes), and tissue engineering (which combines scaffolds, cells, and biologically active molecules to improve or replace biological tissues) [17–24].

Disease-related biomarkers can be rapidly and directly detected by nanostructures, such as nanowires, nanotubes, nanoparticles, cantilevers, microarrays, and nanoarrays, as part of an accurate process characterized by lower sample consumption and considerably higher sensitivity [25–35]. Not only does nanotechnology enable the early identification of viruses, bacteria, and circulating tumor cells, it also has proven potential for single-cell analysis [36].

The use of carbon nanotubes, magnetic nanoparticles, and quantum dot-based nanoprobes for in vivo targeted imaging might pave the way for the rapid, minimally invasive, and more rigorous diagnosis of diseases like cancer, which could be detected at an early stage and monitored throughout its course [37–39]. Moreover, the confirmation of in vivo therapeutic efficacy, the assessment of nanocarrier biodistribution, the ability to pinpoint the exact location of the tumor and the surrounding healthy tissue, and the possibility of lymphatic mapping and sentinel node analysis are other benefits of nanotechnology-based applications [40–44].

Nanomaterials have unique physical and chemical properties (ultra-small size, large surface-area-to-mass ratio, high reactivity) that are different from bulk materials of the same composition and that help them overcome some of the limitations of traditional therapeutic and diagnostic agents [35, 45–48]. Synthesized hafnium oxide-gold core–shell nanoparticles showed enhanced plasmon absorption bands, while synthesized gold nanoshells, gold nanorods, carbon nanotubes and magnetic nanoparticles have been shown to be efficient in nanoparticle-induced magnetic hyperthermia treatments, causing cancerous cells to undergo apoptosis in direct response to applied heat. There are also data demonstrating that nanocrystalline silver dressings may reduce bacterial levels, decrease the chronic inflammatory response, and promote wound healing [17, 18, 49, 50]. There is a need for accurate techniques for pathogenic bacteria identification and detection to allow the prevention and management of pathogenic diseases and to assure food safety.

Conventional techniques employed for detecting microorganisms, such as microbial cultures and bacterial growth, are lengthy and difficult to perform, requiring 6–24 h for the growth process [51], followed by 1–3 days for the morphological and biochemical characterization of bacterial isolates.

The size and properties of nanoparticles make them excellent platforms for the detection and identification of pathogens in native biological samples [16, 28, 52–54]. Magnetic nanoparticles are used for detecting bacteria, which can be further studied using nuclear magnetic resonance imaging [55]. Therefore, the focus of this review is on the current nanoparticle-based techniques for pathogenic bacterial identification and detection using these applications (Table 1).

**Table 1:** The main features of the most important reported methods for bacteria detection

No.	Reference	Type of nanoparticle	Size (nm)	Bacteria detected	Method	Advantages	Limit of detection (CFU/mL)
1.	Joo 2012, [56]	Superparamagnetic Fe_3_O_4_ nanoparticles functionalized with monoclonal antibodies toward Salmonella	120	*Salmonella*	Immuno-magnetic separation	Rapid, and cost-effective	*100*
2.	Wang 2016, [54]	Polyethylenimine (PEI)-modified Au-coated magnetic microspheres (Fe_3_O_4_@Au@PEI) and concentrated Au@Ag nanoparticles (NPs),	300	*Escherichia coli* *Staphylococcus aureus*	SERS detection method	Simple operating procedure, total assay time 10 min.	*100*
3.	Qi 2016, [57]	Cadmium sulfide (CdS) nanoparticles	40–50	*Desulforibrio caledoiensis*	Fluorescence microscopy	Short detection time	*25.8*
4.	Gao 2006, [58]	FePt@Van magnetic nanoparticles,	<10	*Escherichia coli* *Coagulase*-*negative* *Staphylococcus (CNS)*	Fluorescence microscopy	Bacteria detection under 2 h	*4*
5.	Raj 2015, [59]	Cysteine gold nanoparticles (CAuNPs)	20 ± 2	*Escherichia coli*	Colorimetric method	Fast, visual method	*100*
6.	Li 2013, [14]	Streptavidin coated magnetic nanoparticles	36	*Escherichia coli Salmonella* *Vibrio cholera Campylobacter jejuni*	Multiplex PCR	Simultaneous detection of four pathogens	*100*
7.	Cao 2011, [60]	Bimetallic Au@Ag core–shell structures	27.7 ± 6.8	*Campylobacter jejuni*	Immuno-magnetic separation-polymerase chain reaction (IMS-PCR) method	Cost-effective, only basic equipment needed	*100*
8.	Sepunaru 2015, [61]	Silver NPs (AgNPs)	90.4 ± 3.6	*Escherichia coli*	Anodic particle coulometry technique	Single bacteria detection	*Single detection*
9.	Wang 2016, [62]	Au-coated magnetic nanoparticles (AuMNPs) conjugated with *Staphylococcus aureus (S.aureus)* antibody	190	*Staphylococcus aureus*	SERS detection method	Low limit of detection	*10*
10.	Cao 2014, [63]	Molecular beacon–Aunanoparticle	15	*Escherichia coli*	Real-time PCR	10^3^ times more sensitive than traditional beacon probes	*100*
11.	Zhang 2012, [64]	Multifunctional magnetic–plasmonic Fe_3_O_4_–Au core–shell nanoparticles (Au-MNPs)	248.6 ± 35.8	*E. coli* *P. aeruginosa* *A. calcoaceticus*	SERS detection method	One-step concentration and detection	*50*
12.	Wang [65]	CdSe/ZnS@SiO_2_–NH_2_ nanoparticles	70	*Salmonella typhimurium,* *Escherichia coli* *Staphylococcus aureus*	Fluorescence microscopy	High sensitivity	*330*
13.	Zhou 2014, [36]	Silver nanoparticles	30	*Escherichia coli* *Staphylococcus epidermidis*	Dynamic SERS	Total assay time of 10 min.	*250*
14.	Wu 2014, [66]	Multicolor upconversion nanoparticles coupled with magnetic nanoparticles	20−30	*Staphylococcus aureus* *Vibrio parahemolyticus, and Salmonella typhimurium*	Multiplexed Luminescence Bioassay Method	High specificity, simultaneous detection	*10*-*15*

Magnet of luorescent nanoparticles. ACS Nano, 2011. 5(11): p. 8834-8841 [79]. Copyright 2011 American Chemical Society

### Bacterial detection using gold nanoparticles

Gold nanoparticles (AuNP) have been used over a wide range of biological applications due to their characteristics such as the surface Plasmon band localization in the visible spectrum, ease of synthesis, various functionalization capabilities [67–70].

AuNP fluorescent molecular beacons were prepared for synthetic DNA and bacterial 16S rRNA detection of *Escherichia coli* (DH5α) in cell cultures [63]. AuNP–DNA molecular beacon conjugates determined a sensitivity enhancement by three orders of magnitude, allowing for the detection of *E. coli* within 1 h (100 CFU/mL, 1000 times more sensitive than molecular beacons alone). In another instance, dipicolinic acid, a unique biomarker of bacterial spores, was detected by using Tb^3+^ and Eu^3+^ chelated AuNPs as nanosensors [71]. Also, Jin et al. [72] used lanthanide-doped upconversion nanoparticles functionalized with complementary DNA in conjunction with aptamer functionalized gold nanoparticles for a detection platform based on fluorescence resonance energy transfer in order to detect *E. coli* in real food and water samples.

The interaction of AuNPs with cysteine was tested by Raj et al. [59] for the detection of *E. coli*. The binding affinity of *E. coli* 0157:H7 to H2 receptors on the surface of cysteine-modified AuNPs was demonstrated by the electrostatic adhesion between the positively charged cysteine and the negatively charged lipopolysaccharides on the bacterial outer membrane. The negative charge enabled *E. coli* stabilization. The cross-linking of cysteine-capped AuNPs was performed in the presence of a defined number of bacteria with a clear red-to-blue shift in sample color and a highly sensitive naked-eye detection of bacteria. Urine samples (3 × 10^4^ cells/mL) were concentrated using cysteine-capped AuNPs for further clinical use. There was a maximum shift of 10 nm, consistent with values obtained in conventional aqueous solutions, demonstrating that the new method can be used to accurately detect and assess *E. coli* 0157:H7 bacteria. A novel aptasensor for the colorimetric detection of *Salmonella typhimurium* based colour change effect of gold nanoparticles was developed by Ma et al. [73]. Gold nanoparticles were functionalized with aptamers capable recognize *S. typhimurium* which acted as stabilizers for the GNPs even at high concentrations of NaCl. In the presence of the *S. typhimurium* bacteria the aptamers present on the nanoparticle surface bind to the bacteria which results in the aggregation of the GNPs, thus a visible colour change. The limit of detection for this method was as low as 56 CFU/mL. Another study that utilizes the antibody functionalized AuNPs for detection of *Lactobacillus* spp. and *Staphylococcus aureus* was presented by Verdoot et al. [74]. In this case the local surface plasmon resonance (LSPR) band of the AuNPs is monitored by means of UV–Vis spectroscopy.

Polyethylenimine (PEI)-capped AuNPs were developed as another biosensor for the rapid detection of bacteria [75]. The colorimetric assay demonstrated the high binding affinity of positively charged PEI-AuNPs to bacteria with a negative charge. There was a strong interaction between PEI-AuNPs and β-galactosidase on the bacterial surface. The method was conceived to be simple and quick, and allowed a detection limit close to 10 CFU/mL. The aim was to ensure that the positively charged AuNPs selectively bind to the negatively charged bacterial cell wall in the presence of teichoic acids in Gram-positive bacteria or of lipopolysaccharides in Gram-negative bacteria. There were competitive interactions between PEI-AuNPs and the bacterial or enzyme surfaces when β-galactosidase was present.

**Fig. 1:**
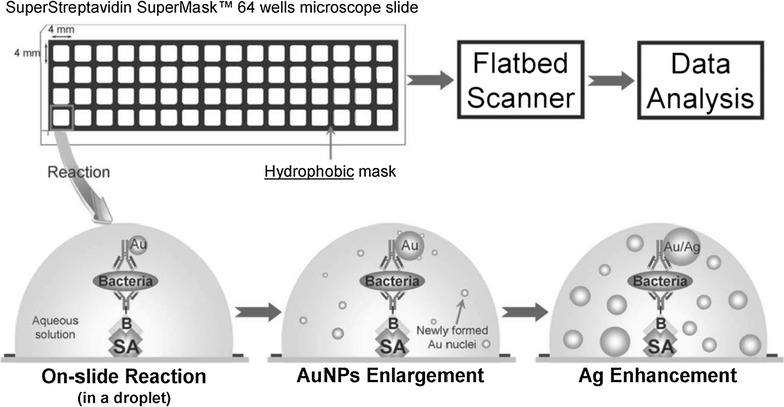
Overall scheme illustrating the procedure of the dual-enhanced scanometric assay for the detection of bacteria (Reprinted with permission from [60])

Bimetallic silver–gold clusters were obtained through synthesis of gold nanoparticle (AuNP) seeds that catalyzed the reduction of gold salts, enhancing the nanoparticles and supplying more gold nuclei with the subsequent addition of silver atoms on AuNPs [60]. The synergistic enhancement effects of a dual catalyst include improved optical intensity that enhances color perception and sensitivity (Fig. 1).

Dual nanocatalysis was employed for the detection of *Campylobacter jejuni*, a Gram-negative spiral-shaped bacteria responsible for campylobacteriosis, one of the most common bacterial infections in humans. The model immunoassay was performed on a glass slide, displaying rapid and selective detection of *C. jejuni* with at least four orders of magnitude greater linear dynamic range and a limit of detection of 10 CFU/mL [60].

Au–Ag core–shell nanoparticles were used by Ding et al. [76] to form aggregates for simultaneous bacterial imaging and synergistic antibacterial activity. The principle of this approach is based on the fact that the positively charged Au–Ag core–shell NPs form aggregates on the negatively charged bacterial surface of *S. aureus* with an enhanced two-photon photoluminescence effect.

Gold-coated magnetic nanoparticles were synthesized employing hydroxylamine seeding based on a sonochemical method [62]. The nanoparticles had the same size and shape, were stable, and possessed excellent magnetic responsivity and strong surface-enhanced Raman spectroscopy (SERS) activity.

**Fig. 2:**
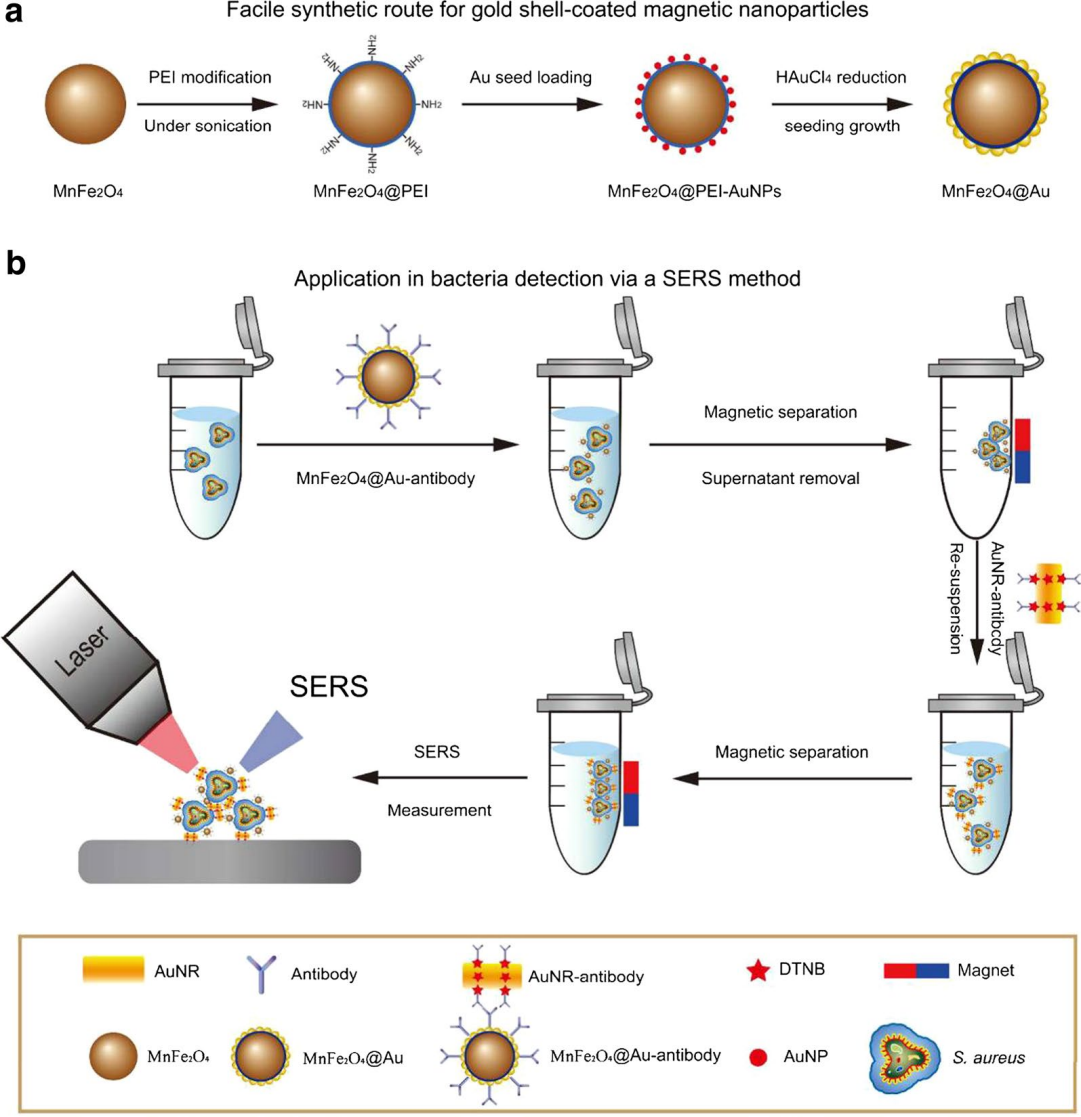
**a** Schematic representation of the synthesis of gold-coated magnetic nanoparticles. **b** Schematic representation of the methods employed for the detection of bacteria using a SERS method (Reprinted with permission from [62]. Copyright 2016 American Chemical Society)

A highly sensitive sandwich-structured SERS platform was developed for the detection of *S. aureus*. A SERS assay and a SERS tag enabled the bio-separation and detection of bacteria. There were strong coupling interactions between AuMNPs and the bacterial samples, with multiple hotspots and an increase in the SERS signal. Individual and inter-particle magnetic hotspots identified an increase in the Raman signal of the bacterial samples and a subsequent higher sensitivity of bacterial detection. *S. aureus* was detected via the SERS method based on the sandwich-structured immunoassay, with a detection limit of 10 cells/mL (Fig. 2).

A label-free method for the quick and effective detection of bacteria was developed in another study using SERS, PEI-conjugated gold-coated magnetic microspheres, and gold-shell-coated silver nanoparticles (AgNPs) [54]. The capture-enrichment-enhancement (CEE) three-step method employed the capture and enrichment properties of PEI-conjugated gold-coated magnetic microspheres and SERS enhancement properties, so that the bacteria could be detected within 10 min. The synthesis of magnetic microspheres for the efficient capture and separation of bacteria and for use as SERS substrates for bacterial analysis, and that of concentrated gold-shell-coated AgNPs for their reinforcing efficiencies, was consistent with the method requests. There were strong electrostatic interactions between PEI containing a positive charge and bacterial cells with a negative charge, which allowed for rapid and efficient bacterial capture and enrichment. This method proved to be efficient when using the optimum incubation time and particle concentration, detecting *E. coli* and *S. aureus* at a minimum detectable level of 1000 CFU/mL.

**Fig. 3:**
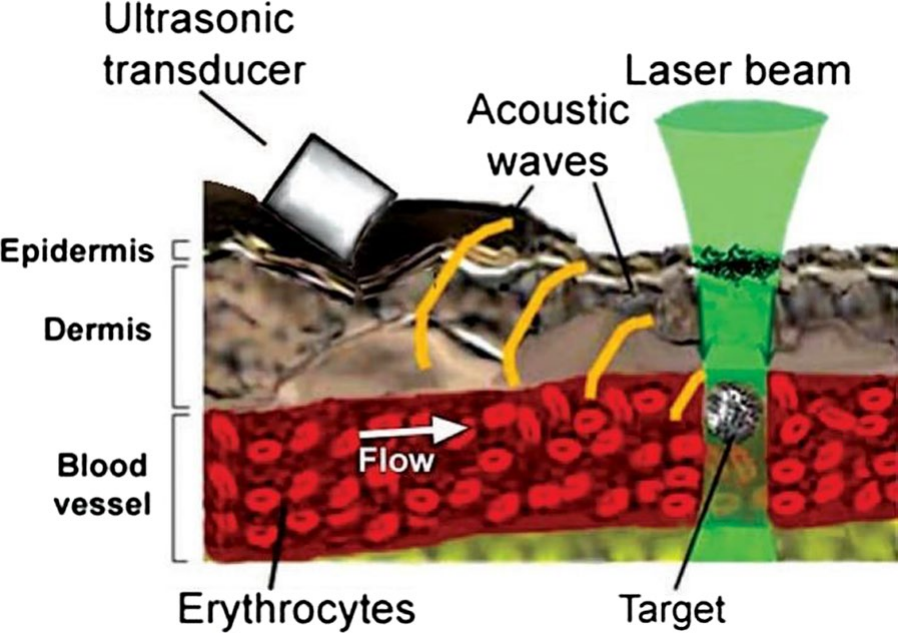
Photoacoustic in vivo detection of single absorbing targets in circulation (Reprinted with permission from [73])

The photoacoustic detection of bacteria demonstrated potential in the peripheral blood of mice and rats (10^2^ bacteria every 15 min), also exhibiting a possible increase [77]. The photoacoustic flow cytometry method was developed for in vivo detection of single absorbing targets in circulation based on time-resolved monitoring, with or without nanoparticles as photoacoustically detectable labels (Fig. 3). This was the first study to prove the potential of photoacoustic flow cytometry with tunable near-infrared (NIR) lasers for the real-time monitoring of gold nanorods, *S. aureus* and *E. coli* labeled with carbon nanotubes, and Lymphazurin in the ear blood microvessels and mesenteric structures of mice and rats. This method allows detection of single circulating cells or bacteria in the entire circulation of mice during continuous photoacoustic monitoring of larger vessels every hour. The results indicated a clearance rate with ultra-high sensitivity for a single bacterium (or cancer cell) in a background of 10^8^ normal blood cells.

### Bacterial detection using silver nanoparticles

Silver nanoparticles (AgNPs), due to their unique characteristics, present a high interest for several applications in life sciences. For example, Raman-based detection of bacteria using protein-A-antibody-modified AgNPs was employed in a study conducted by Ghinwa et al. [78]. Selectivity was achieved by incubating bacteria with adequate polyclonal antibodies. The selectivity of bacteria with surface-enhanced Raman spectroscopy was superior to that of bulk Raman spectroscopy.

**Fig. 4:**
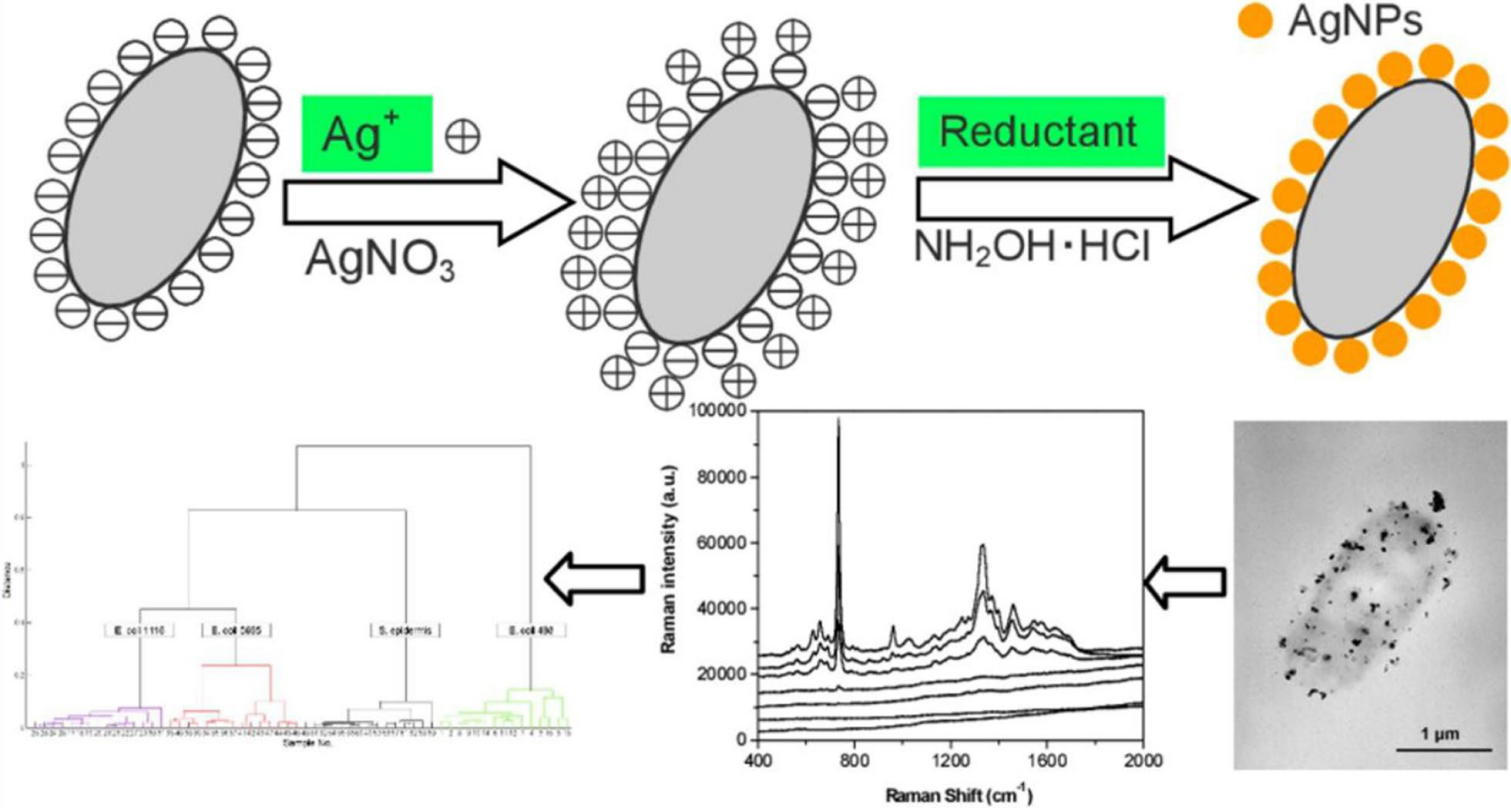
In situ synthesis of AgNPs on the surface of bacteria, showing the electrostatic attraction between the nanoparticles and their cellular targets following the addition of the reducing agent (Reprinted with permission from [36]. Copyright 2014 American Chemical Society)

The surface charge of the cell wall was mostly responsible for the Raman spectra of microorganisms upon in situ synthesis of AgNPs directly on the surface of bacteria [36] (Fig. 4). This method showed that the Raman signal of these bacteria was approximately 30 times higher than that obtained by mixing colloid and bacterial suspensions. The total time to set up and complete the assay was only 10 min and the total volume of the reactants required to assess the bacteria was 1 mL. As little as 3 μL of sample was enough to perform the SERS measurements. Moreover, this new approach described by the authors was able to differentiate between three *E. coli* strains and one *Staphylococcus epidermidis* strain by means of a hierarchy cluster analysis. SERS mapping detected 250 CFU/mL on hydrophobic glass slides.

Lectin-sensitized anisotropic AgNPs were prepared to detect various bacterial strains, both Gram-negative and Gram-positive [79]. Major changes in the optical properties of the nanoparticles were induced following their interactions with bacteria.

Individual bacteria were rapidly detected using anodic particle coulometry [61]. As their binding affinity is well-known, citrate-capped AgNPs (9.4 ± 3.6 nm diameter) and *E. coli* demonstrated the detection of individual bacteria within 1 min in solution containing 0.3 pM *E. coli* cells. Due to factors such as the high surface functionality of bacteria, the affinity of AgNPs for bacteria, and the electrochemical properties of the nanoparticles, the charge of individual bacteria was twice as high as the noise level.

Sulfate-reducing bacteria were selectively detected in a new approach using the photocatalytic properties of zinc sulfide (ZnS) nanoparticles [80]. The synthesis of these nanoparticles was based on specific bacterial metabolism and they were employed as photocatalysts for the photodegradation of methylene blue. This process was influenced by the baseline concentration of bacteria. There was a linear relationship between the photodegradation of methylene blue and bacterial concentration (1.0 × 10^3^ to 1.0 × 10^8^ CFU/mL). The new approach also showed good specificity for the detection of sulfate-reducing bacteria.

An assay based on surface-enhanced Raman spectroscopy analysis and AgNPs was developed to detect individual bacterial species on microarrays [81]. SERS-active AgNPs were synthesized directly onto the surface of bacterial cells, mainly *E. coli*, determining the high detection-sensitivity of these microorganisms on a microarray platform. This method also employed receptor-selective antibodies. There was a tenfold increase in the Raman signal of the novel nanocompound compared to the values obtained when simply combining bacteria with AgNPs. Laser excitation below 633 nm exhibited optimal SERS activity of bacteria@AgNPs, probably as a result of the surface plasmon interactions of aggregated AgNPs.

### Bacterial separation/detection using magnetic nanoparticles

Nanoparticle-based platforms have been employed for the diagnosis of tuberculosis in order to achieve early detection for better infection control. Bacillus Calmette-Guérin (BCG) was used as a surrogate for *Mycobacterium tuberculosis*, showing a remarkable detection speed and sensitivity. Twenty CFU/mL were detected in sputum in <30 min [82].

Fluorescent magnetic nanoparticles have been used for biomedical applications to achieve rapid, sensitive, and cheap detection of bloodstream infections. Vancomycin-modified magnetic nanoparticles were used to generate multivalent interactions and to detect bacteria in blood samples [58]. The enrichment culture was further subjected to fluorescent vancomycin staining and bacteria were detected within 2 h, showing a sensitivity of 10 CFU/mL. The back titration method was used to confirm bacterial counts.

**Fig. 5:**
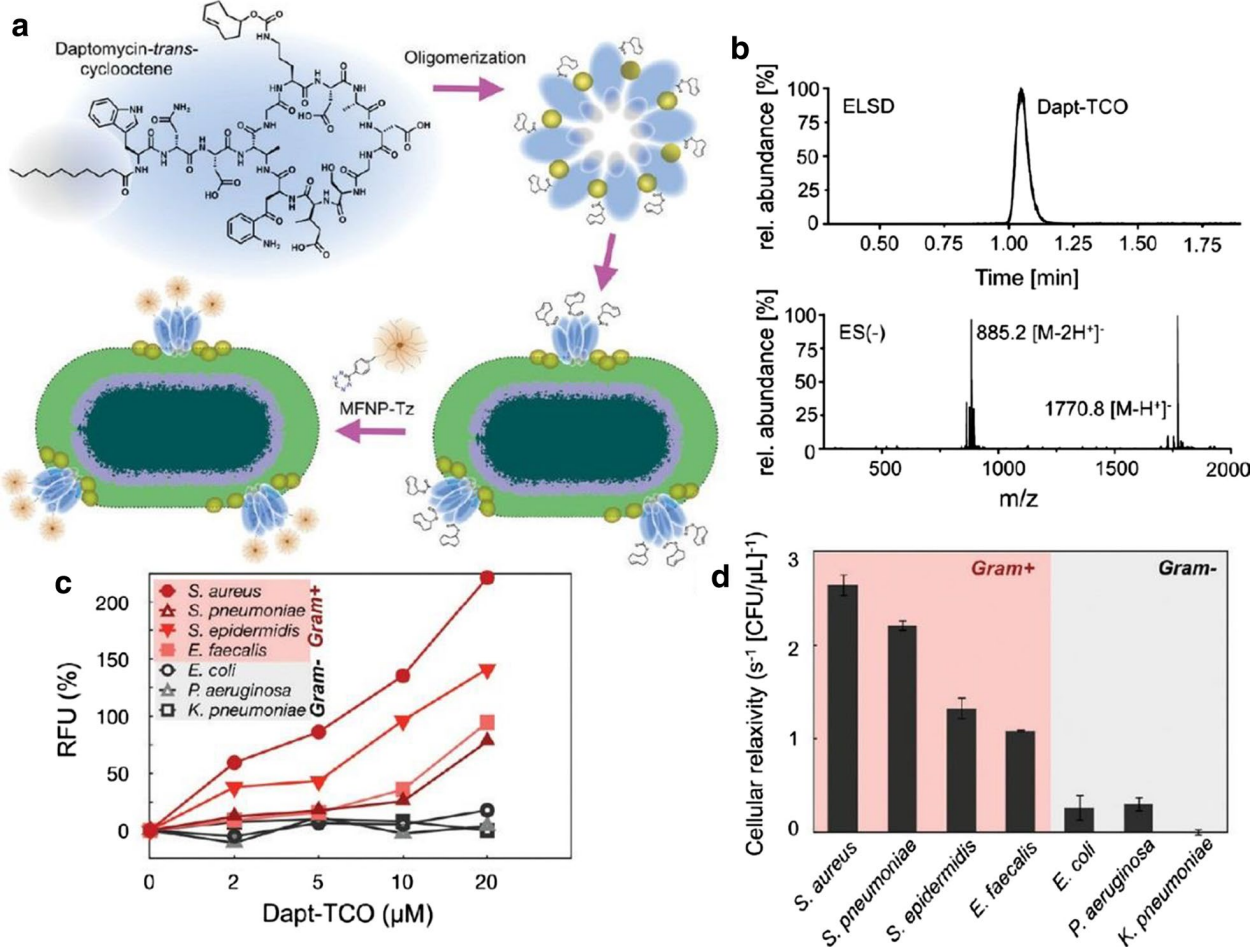
**a** Representat ion of trans-cyclooctene (TCO) derivatives of daptomycin binding to Gram-positive bacteria. Ca2þ channels control the process of oligomerization and binding to the cell surface results in the format ion of pores in the peptidoglycan layer. **b** Dapt-TCO derivatives detected using high-performance liquid chromatography and elect rosprayionization mass spectrometry. **c** Fluorescence spectrometry of bacterial cells stained with various concent rations of Dapt-TCO and labeled with magnet of luorescent nanoparticles (data expressed as mean and standard deviation). **d** Bacteria tracking by magnetic resonance (data expressed as mean and standard error) (Reprinted with permission from [79]. Copyright 2011 American Chemical Society)

Other researchers have shown that the antibiotic vancomycin modified with trans-cyclooctene (Vanc-TCO) binds to the cell wall of Gram-positive bacteria by forming hydrogen bonds with the terminal d-alanyl-d-alanine (d-Ala-d-Ala) moieties of the *N*-acetylmuramic acid (NAM) and *N*-acetylglucosamine (NAG) peptide subunits [83]. Magnetofluorescent nanoparticles attached to tetrazine (MFNP-Tz) were employed for bacterial labeling using bioorthogonal chemistry. The results showed dose-dependent binding of Vanc-TCO to *S. aureus*, *S. pneumoniae*, *S. epidermidis*, and *E. faecalis* cell walls. Therefore, labeling of Gram-positive bacteria can be achieved using bioorthogonal magnetofluorescent nanoparticles, also enabling the optical and magnetic detection of bacteria (Fig. 5). There was concentration-dependent binding of nanoparticles to bacteria, varying according to the type of bacteria and inhibited with unmodified antibiotics [83].

It was also demonstrated that the bioorthogonal labeling of living bacteria using synthesized TCO derivatives of vancomycin resulted in enhanced antibacterial activity following incubation for 24 h. This phenomenon might be caused by interference with the peptidoglycan layer in the bacterial cell wall, which is formed from the alternating linear chains of NAG and NAM amino sugars. The permeability of Vanc-TCO into mammalian cell cultures might also enable the detection of intracellular bacteria, for example those within macrophages.

**Fig. 6:**
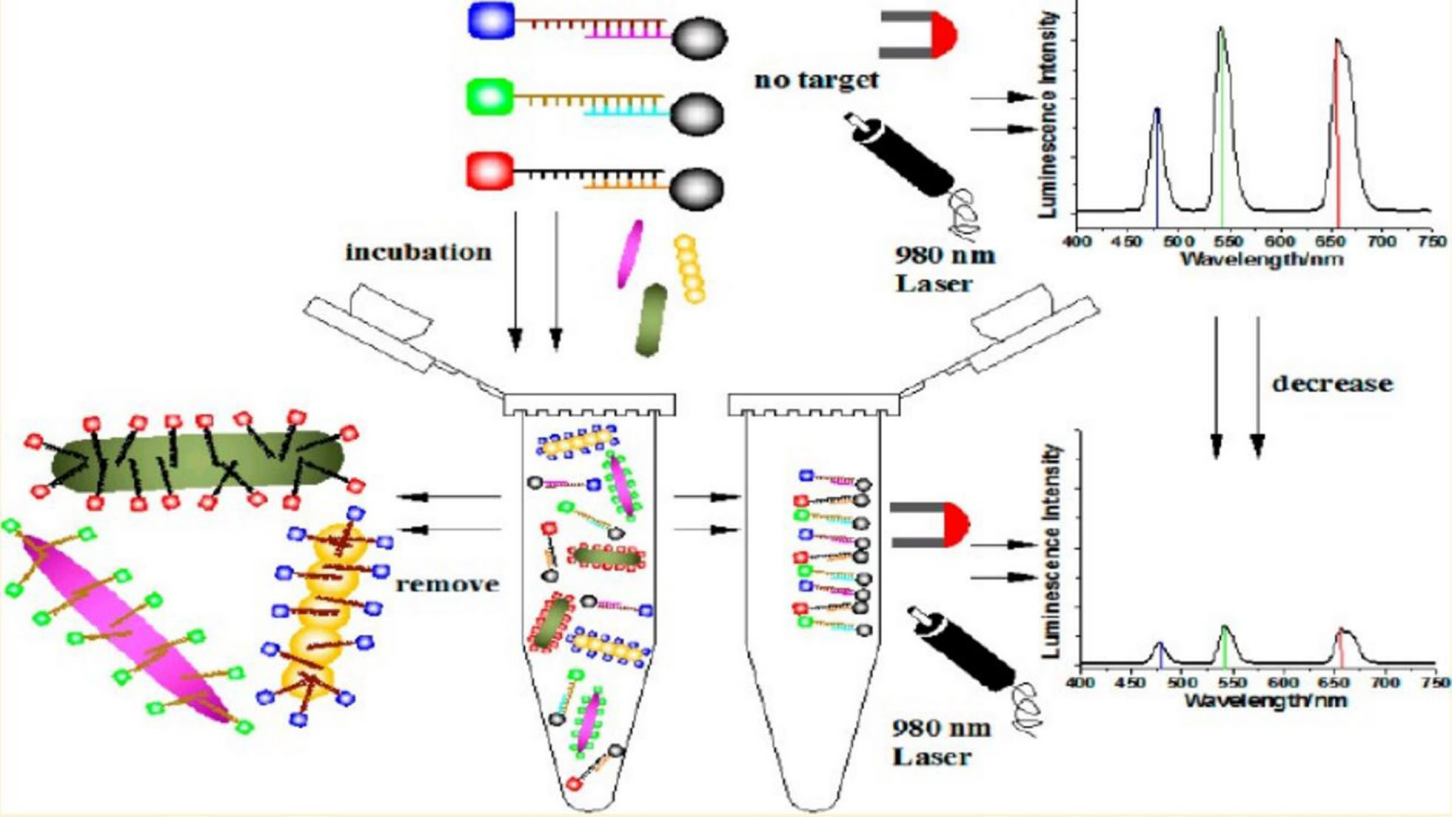
Graphical representation of bacterial detection based on a multiplexed fluorescence resonance energy transfer aptasensor (Reprinted with permission from [66]. Copyright 2014 American Chemical Society)

Bacterial detection based on multicolor upconversion nanoparticles coupled with magnetic nanoparticles was employed using a multiplexed fluorescence resonance energy transfer aptasensor (Fig. 6). The targeted bacteria were *S. aureus*, *Vibrio parahaemolyticus*, and *S. typhimurium* [66]. Bacterial detection was achieved using different rare-earth-doped upconversion nanoparticle labels with independent emission peaks. Autofluorescence of biomolecules was prevented using a 980 nm infrared diode laser. Efficient separation and concentration of targets from interferences in the food matrix were accomplished by magnetic nanoparticles, with no need to pretreat the samples. Additionally, this method resulted in stable and target-specific aptamers, superior to the susceptibility of traditional antibodies. The authors reported the benefits of a novel aptamer-based detection assay used as a stable bioassay platform that provides high sensitivity and specificity [66].

Applications based on fluorescent nanocrystal quantum dots were described for the detection of bacteria with flow cytometry and flow cytometric determination of magnetic nanoparticle collection and separation [84]. The authors employed quantum dot fluorescent labels for specific conjugates against *Bacillus anthracis* and *Yersinia pestis*, facilitating bacterial dissemination. Highly sensitive multiplex detection of the two types of bacteria was achieved (10^3^ bacteria/mL in the original sample). Fluorescence-activated cell sorting (FACS) was used for sensitive and selective analysis and enumeration of viable bacteria in the sample. The authors also developed an assay for the collection of viable Gram-negative bacteria (*Y. pestis*). Magnetic nanoparticles enabled the immunomagnetic detection and separation of bacteria, with fast sorting at high/low bacterial concentrations (10^**5**^ CFU/mL) [84].

A magnetic nanoparticle microarray was developed for the simultaneous and simple detection of foodborne pathogens [14]. DNA microarray analysis using multiplex polymerase chain reaction (PCR) and labeling based on magnetic nanoparticles was described by Song et al. for the simultaneous detection of four common foodborne pathogens, *E. coli* O157:H7, *Salmonella enterica*, *Vibrio cholera*, and *C. jejuni*. The process employs two-stage amplifiers to simultaneously intensify target genes. The hybridization of biotinylated single-stranded PCR products to microarray probes was followed by the addition of magnetic nanoparticles coated with streptavidin in order to visualize the results of the hybridization process. The assay proved efficient and exhibited low detection limits (316 CFU/mL), suitable for diagnosing infectious diseases in the laboratory or as part of real-time observations.

Sequence-specific detection of individual DNA strands was achieved in an assay developed by Strelau et al. [85] using surface-enhanced Raman scattering and magnetic nanoparticles for enrichment of target molecules. Hybridization in solution was employed for rapid and efficient binding of longer DNA strands (PCR products). DNA strands were purified and amplified after they were separated using magnetic nanoparticles. The hybridization of a dye-modified, short synthetic single-stranded DNA was performed upon binding of the target DNA, serving as a Raman label. Molecular binding was detected by SERS spectra. The method was first applied using short synthetic oligonucleotides to assess its specificity, and later used to detect PCR amplification products for specific identification of epizootic hemorrhagic disease viruses. The authors used PCR targets based on sequences of *Mycoplasma mycoides* subspecies *mycoides* Small Colony type, which causes contagious bovine pleuropneumonia. Three different PCR products labeled with three different dyes were simultaneously detected to show the strong SERS multiplexing ability. The results indicated the likelihood of employing magnetic beads for Raman-based detection of PCR products amplified from the DNA of epizootic pathogens.

The synthesis of amino-functionalized silica-coated magnetic nanoparticles and PCR was used for the rapid detection of foodborne pathogens via DNA isolation from milk [55]. The rapid and sensitive detection of single or multiple target pathogens was achieved in combination with PCR identification. The method employed both Gram-negative (*Salmonella enteritidis*) and Gram-positive (*Listeria monocytogenes*) bacteria to contaminate raw milk samples, obtaining a detection limit comparable to that of the immunomagnetic separation-polymerase chain reaction (IMS-PCR) assay. The detection limit in artificially contaminated raw milk was 8 CFU/mL for *S. enteritidis* and 13 CFU/mL for *L. monocytogenes* when using the simplex PCR assay, and 15 CFU/mL for *S. enteritidis* and 25 CFU/mL for *L. monocytogenes* when using the multiplex PCR assay. *Listeria monocytogenes* detection by employing magnetic nanoparticles (MNPs) and the NMR technique was proposed by Zhao et al. [86] Antibody functionalized silica coated MNPs dispersed in solution were found to self-assemble on the surface of a bacterial target accompanied by the increase of the T2 value of water protons which is detected with the aid of nuclear magnetic resonance, with a total protocol time of 40 min.

In another study, pathogenic bacteria were detected using antibody-conjugated magnetic nanoparticles [87]. Following synthesis, nanoparticles were magnetically separated from the analyte and used to detect *Salmonella* in milk. The MNP-*Salmonella* complexes were redispersed in buffer and exposed to antibody-immobilized TiO_2_ nanocrystals with increased light absorption near 230 nm. Following magnetic separation, the UV–Vis absorption spectra were collected for the solutions containing unbound TNs. Due to the inversely proportional relationship between light absorption intensity and the concentration of bacteria, there was high sensitivity for low concentrations of *Salmonella*. The results obtained a detection limit of *Salmonella* in milk samples of >100 CFU/mL.

**Fig. 7:**
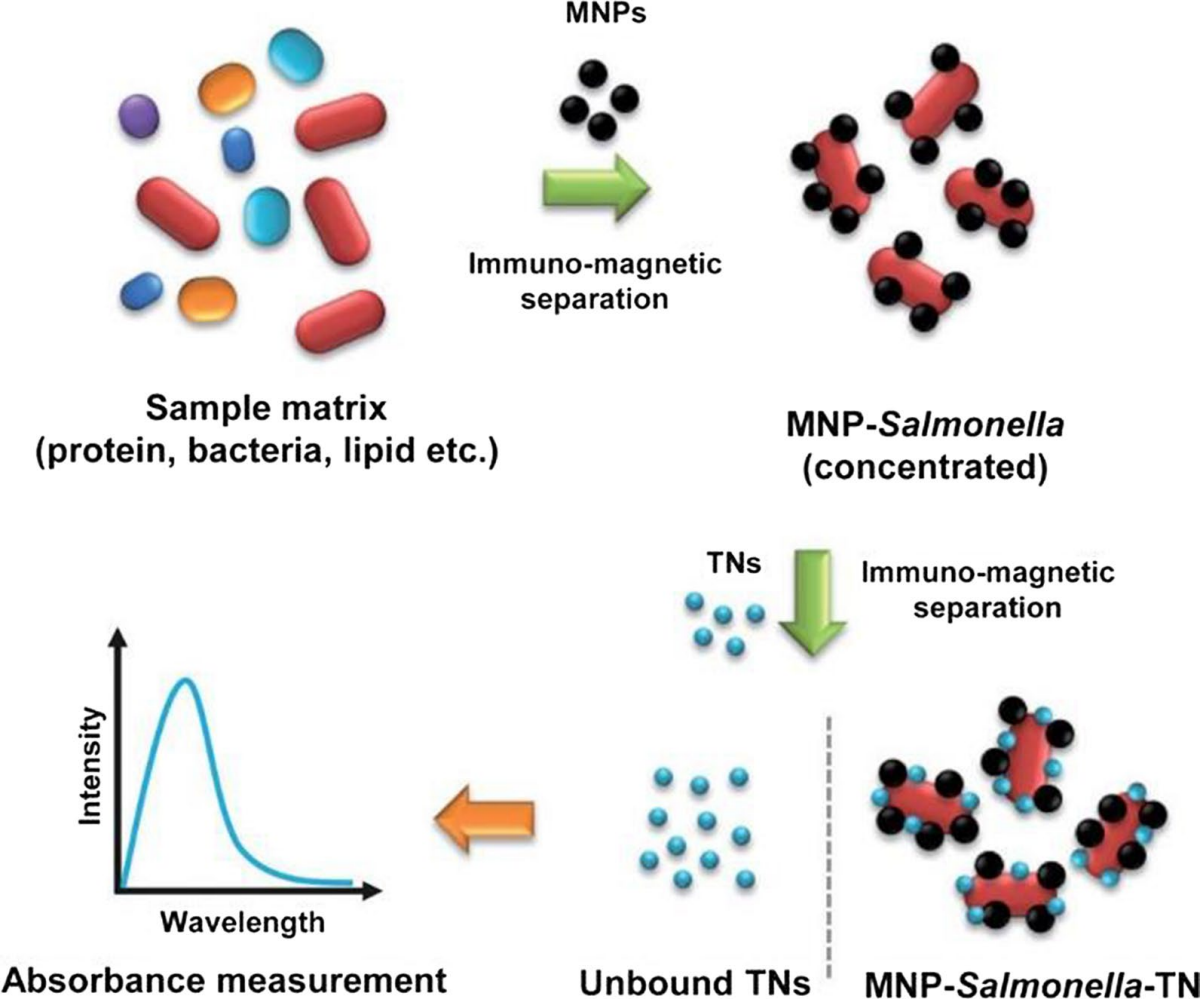
Representation of the method employed for the detection of pathogenic bacteria using magnetic nanoparticles and optical nanoprobes (Reprinted with permission from [56])

The quick, easy, and inexpensive detection of *Salmonella* in buffer solution or in a milk matrix was achieved in a study conducted by Joo et al. [56] based on magnetic nanoparticles and titania (TiO_2_) nanocrystals by isolating cells using immunomagnetic separation alongside optical sensing. Quick detection of bacteria was enabled by magnetic nanoparticles under external magnetic fields. Titania nanocrystals were employed as optical nanoprobes for spectroscopy assessment, as they display better spectral stability than AuNPs at a range of pH values, temperatures, and salt concentrations. The conjugation of antibodies to magnetic nanoparticles can selectively target bacteria after being separated from solution by the application of an external magnetic field. There was an increase in absorbance at 230 nm following the binding of antibody-conjugated titania nanocrystals to the MNP-Salmonella complexes. The authors reported bacterial detection limits of >100 CFU/mL in milk (Fig. 7).

Synthesized core–shell gold-coated Fe_3_O_4_ magnetic nanoparticles (AuMNPs) were characterized using SERS for simultaneous fast concentration and sensitive detection of bacteria [64]. The application of an external magnetic field resulted in effective condensation within 5 min using 10 µL of 10^5^ CFU/mL bacteria and 3 µg/mL AuMNPs on a silicon surface. The concentration of bacteria in the dot area was 60 times greater than in the surrounding area. The bacteria were surrounded by homogeneous AuMNPs, which enabled their sensitive and reproducible detection by SERS. Three different strains of Gram-negative bacteria could be distinctly discriminated when using the principle component analysis (PCA) technique. The authors indicated the potential use of quantum dot AuMNPs as highly sensitive SERS-active substrates with a detection limit that was better than 0.1 ppb for small molecules (for example, 4-mercaptopurine).

In another study, quaternized magnetic nanoparticles (q-MNP)–fluorescent polymer systems were employed in an assay to support bacterial identification and detection [88]. The authors showed that the kinetics of q-MNP-PFBT polymer conjugates suggests their use as competent sensors for detecting pathogens. The application of linear discriminant analysis (LDA) allowed the successful classification of eight different bacterial species: *Shewanella oneidensis*, *Vibrio fischeri*, *Micrococcus luteus*, *Edwardsiella tarda*, and *E. coli* in Luria–Bertani medium; *Vibrio alginolyticus* and *Pseudomonas aeruginosa* in tryptic soy broth; and *Pichia pastoris* in yeast extract peptone dextrose medium. The canonical discriminant analysis was quick, easy to perform, and inexpensive. The sensitivity of this approach might be suitable for the detection of pathogenic bacteria, proteins, or DNA tests [88].

### Bacterial detection using other types of nanoparticles

The capture and detection of *E. coli* was achieved with immuno-nanorice particles obtained by attaching polyclonal antibodies to the surface of the nanorice (hybrid nanoparticles with plasmonic properties of both nanoshells and nanorods in a single structure). The attachment of specific anti-*E. coli* antibodies to the nanorice was aided by protein-A molecules. The new particles demonstrated their binding affinity for *E. coli* cells. Following the capture of bacterial cells, the immuno-nanorice-bacteria complex was separated from the aqueous solution using the magnetic properties of the nanorice. Ultraviolet resonance Raman (UVRR) spectroscopy allowed the detection of single bacterial cells when analyzing the bacteria sorbed onto the immuno-nanorice, with the substrate increasing the Raman intensity by several orders of magnitude [89]. In another instance, the differentiation between Gram+ and Gram− bacteria was achieved by using 3D bio-inorganic scaffold of Agnanoplate aggregate–bacteria–Au@Ag vertical nanorod supercrystal coupled with SERS [90].

Another nanosystem for bacterial detection and inhibition was designed using polyion complex (PIC) micellar nanoparticles [91]. The binding of bacteria to the PIC nanosystem resulted in dissolution as a consequence of competitive binding of polycation blocks with negatively charged bacterial surfaces, followed by prominent fluorescence employed as a real-time module for microbial detection. The use of fluorometric assays led to a detection limit of 5.5 × 10^4^ CFU/mL for suspensions of *E. coli* cells. The minimum inhibitory concentration of PIC micellar nanoparticles was 19.7 mg/mL, demonstrating their potential use as antibacterial agents and displaying significant antibacterial effects.

Isothermal amplified detection of RNA was employed for the visual and sensitive detection of viable pathogenic bacteria based on a bioactive paper-based platform [92]. The results were transformed into a two-dimensional bar code for a more thorough analysis. The assay included quick and efficient RNA extraction, amplification, and visualization. Concentrations of viable *L. monocytogenes* exhibited a detection limit of 0.5 pg/μL genomic RNA with high specificity within 15 min. The assay can be employed for the specific detection of 20 CFU/mL of *L. monocytogenes* in milk and cheese samples. The authors demonstrated that due to its viability, this assay allows for the transmission, reception, and sharing of the results with remote examiners for further testing [92].

*Vibrio parahaemolyticus* and *S. typhimurium* were simultaneously detected as part of an approach using a dual fluorescence resonance energy transfer (FRET) system from green-emitting quantum-dots (gQDs) and red-emitting quantum-dots (rQDs) and nanoparticles containing amorphous carbon [28]. The gQDs-aptamer conjugates were employed to detect *V. parahaemolyticus*, while the rQDs-aptamer conjugates detected *S. typhimurium*. There was considerable fluorescence quenching between quantum dots and CNPs. There was also a linear relationship between quantum dot fluorescence emission and bacterial concentration, ranging from 50 to 106 CFU/mL. This method identified *V. parahaemolyticus* with detection limits of 25 CFU/mL, and *S. typhimurium* with detection limits of 35 CFU/mL. Bacteria were detected in real food samples and the results were in agreement with those obtained by the conventional plate count method [28].

Bacterial detection based on the preparation of CdSe/ZnS@SiO_2_ fluorescent nanoparticles was suggested in another study [65]. The bacterium employed in this review was *S. typhimurium*. The reverse micro-emulsion method was used for the preparation of quantum dot-doped silica nanoparticles. The conjugated nanocomposite was prepared based on a two-step procedure for cross-linking with glutaraldehyde. The intensity of fluorescent light was directly proportional to the concentration of bacteria (6.6 × 10^2^ to 6.6 × 10^4^ CFU/mL, equation *I* = 0.1331 log *C* − 0.2017, R^2^ = 0.9974). The results showed a detection limit of 3.3 × 10^2^ CFU/mL. This assay might also be used to detect other types of bacteria.

Cadmium sulfide (CdS) nanoparticles were prepared as fluorescent sensors for detection of sulfate-reducing bacteria (SRB) [57]. The nanoparticles were analyzed by transmission electron microscopy, selected area electron diffraction (SAED), and fluorescence spectroscopy. The CdS nanoparticles demonstrated a linear response in SRB culture solution (1.0 × 10^2^ to 1.0 × 10^7^ CFU/mL). There was a direct relationship between bacterial concentration and the intensity of the spectra, with a greater detection of SRB at higher concentrations. Concentrations ranged from 1.0 × 10^2^ to 1.0 × 10^7^ CFU/mL. There was a regression slope of 104.23 and a correlation coefficient of 0.996 (FI = 104.23 × logN_SRB_ + 30.63). This newly developed method for the detection of SRB might replace biological recognition elements, as it is cheaper, less time-consuming, and able to preserve specific recognition properties. In another study, cadmium tellurium quantum dots were proposed by Cihalova et al. [93] for the detection of *S. aureus*, MRSA and *K. pneumoniae*. By preparing complementary oligonucleotides for the specific bacterial genes (*fnbA* for *S. aureus*, *mecA* for MRSA and *wcaG* for *K. pneumoniae*) and labeling them for simultaneous detection the detection limit of the method was as low as 10^2^ CFU/mL.

## Conclusion

The present review aims to discuss the significance and current issues related to the detection of bacteria, discussing some of the most relevant nanoparticle-based assays for this process. We provide information regarding the various methods employed for the detection of bacteria using vancomycin-, daptomycin-, and antibody-modified nanoparticles, as well as details on the bioconjugation of different types of nanoparticles. The use of nanoparticle-based methods might lead to further advances in the development of highly sensitive and specific assays for bacterial detection.
